# Sensitivity, Specificity, and Limitations of Optical Coherence Tomography Angiography in Diagnosis of Polypoidal Choroidal Vasculopathy

**DOI:** 10.1155/2017/3479695

**Published:** 2017-12-12

**Authors:** Yi-Ming Huang, Ming-Hung Hsieh, An-Fei Li, Shih-Jen Chen

**Affiliations:** Department of Ophthalmology, Taipei Veterans General Hospital, Taipei, Taiwan

## Abstract

**Purpose:**

To evaluate the sensitivity and specificity of optical coherence tomography angiography (OCTA) in differentiating polypoidal choroidal vasculopathy (PCV) from age-related macular degeneration (AMD).

**Methods:**

Fundus color photographs, spectral-domain optical coherence tomography, and fluorescein angiography (step 1) and OCTA (step 2) of 50 eyes that had PCV or AMD were presented to two ophthalmologists. The final diagnoses of PCV were masked. Sensitivity and specificity were calculated and compared to the 2-step approach (before and after OCTA) in detecting PCV. The limitations were also evaluated.

**Results:**

Of the 50 eyes, 31 were PCV and 19 were non-PCV. The sensitivity increased from 69.5% to 90% after OCTA; however, there was no significant improvement in specificity after OCTA. 70.9% of the eyes with PCV had clear or obvious branching vascular nets (BVNs) in OCTA with high sensitivity (97.5%) after OCTA. Contrarily, 29.1% had insignificant BVNs with a low sensitivity (72.5%) after OCTA. 27% of the occult choroidal neovascularization (CNV) cases were overdiagnosed as PCV when OCTA was applied.

**Conclusions:**

OCTA based on clear BVNs at the choroidal level increased sensitivity of diagnosis of PCV by 20%. However, the false-positive rate also increased in occult CNV. Several limitations for a correct diagnosis of PCV were noted.

## 1. Introduction

Idiopathic polypoidal choroidal vasculopathy (PCV) was first described by Yannuzzi et al. and is characterized by recurrent subretinal and subretinal pigment epithelium bleeding [1, 2]. PCV seems to be a distinct clinical entity and differs from other types of choroidal neovascularization in age-related macular degeneration (AMD). It is prevalent in Asia, accounting for about 40% to 50% of cases of AMD [2, 3].

The fundus characteristics of PCV include subretinal red or orange nodules and hemorrhagic or exudative pigment epithelial detachment (PED) [1, 4]. Most eyes with PCV display features similar to occult choroidal neovascularization (CNV) in fluorescein angiography (FA) [5]. Spectral-domain optical coherence tomography (SD-OCT) is another useful tool based on features of sharp PED and double-layer signs at the retinal pigment epithelium [6]. However, indocyanine green angiography (ICGA) is the gold standard tool for the diagnosis of PCV, where polypoidal dilation and choroidal branching vascular nets (BVNs) are observed [7].

OCT angiography (OCTA) is a new imaging tool used to diagnose PCV. The polyps appear as hypoflow round structures, whereas the BVNs are detected as hyperflow vascular networks [8]. However, whether OCTA can help differentiate PCV with various clinical signs that resemble AMD is unknown. [9] Therefore, the aim of this study was to evaluate the sensitivity, specificity, and limitations of OCTA in distinguishing PCV from AMD in clinical practice.

## 2. Patients and Methods

**Figure 1:**
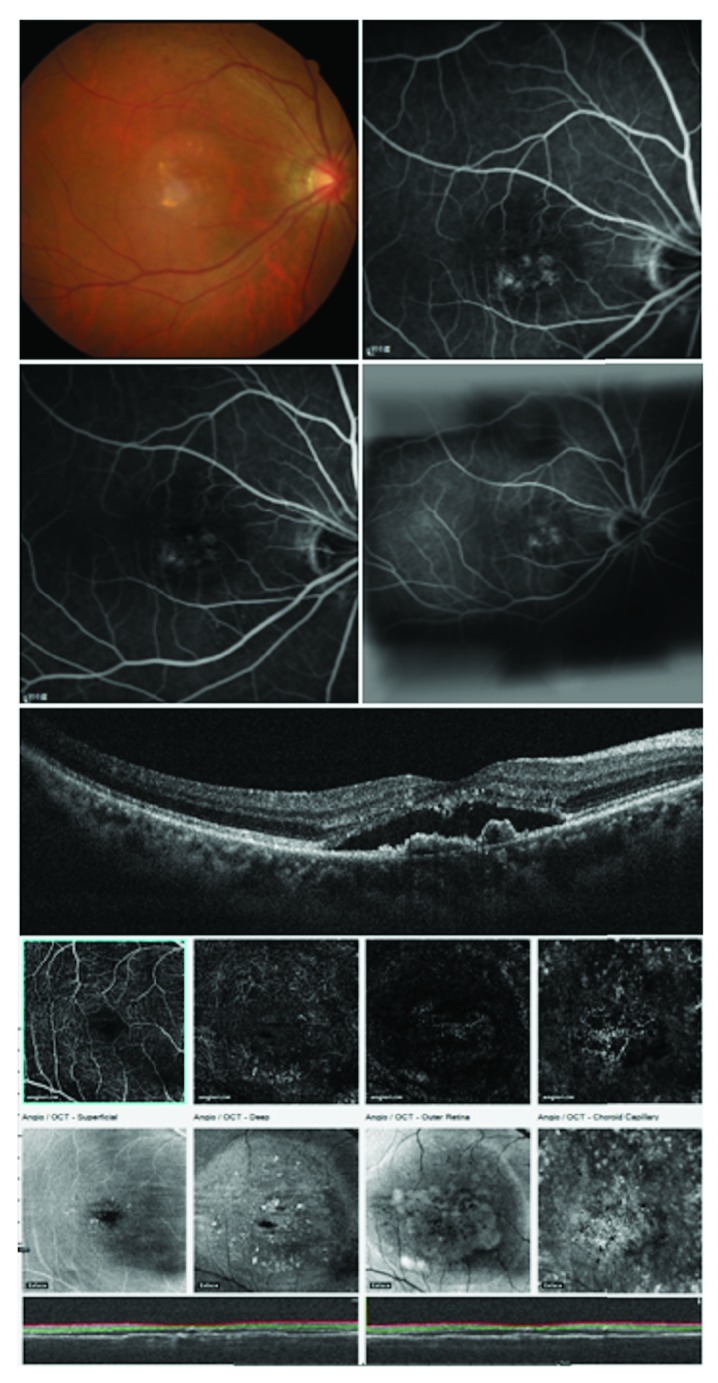
Two steps of multimodal evaluation of an example. In the first step, one color picture of the macula (top left), early (top right), mid, and late phases (the second row, left to right) of fluorescein angiography, and spectral-domain optical coherence tomography images (the third row) of all cases were given to the two graders. In the second step, the optical coherence tomography angiography (OCTA) images including four images at the superficial retina, deep retina, outer retina, and choriocapillary level were provided (the fourth to sixth rows). The two graders then made a diagnosis of polypoidal choroidal vasculopathy (PCV) or non-PCV in each step.

We retrospectively collected images of patients who had macular subretinal fluid with or without pigment epithelial detachment (PED) or hemorrhage attributable to either PCV or AMD (occult CNV, retinal angiomatous proliferation, classic CNV, and drusenoid PED and mixed with both occult and classic CNV) from January 2015 to February 2016 in a tertiary medical center in Taiwan. The images included color fundus photographs, SD-OCT (Optovue, Fremont, CA), OCTA (Avanti; Optovue, Fremont, CA), FA, and ICGA (Heidelberg Engineering Inc., Heidelberg, Germany). Patients with retinal vascular occlusion, myopic CNV, and other secondary CNVs, diabetic retinopathy, and central serous chorioretinopathy were excluded. Poor quality images such as those with a hazy medium or poor fixation of OCTA were also excluded. The patients with a history of treatment including photodynamic therapy (PDT) or intravitreal injections of antivascular endothelial growth factor (VEGF) therapy were not excluded. The OCTA images were not further refined or modified even if autosegmentation was not perfectly aligned, and the cross-sectional picture with the segmentation line was provided (Figure 1, bottom). The diagnosis of PCV was confirmed by ICGA as the presence of polyps with or without BVNs. The eligible images were then masked and tested by one senior retina specialist and one retinal fellow for the diagnosis masked to the results of ICGA.

At the first step, one color image of the macula, three FA images (early, mid, and late phases), and two (horizontal and vertical) cross-sectional SD-OCT images of all cases were provided to the two graders (Figure 1) who were then asked if the diagnosis was PCV or non-PCV. Cases that could not be determined with the provided images were classified as being non-PCV. In the second step, the graders were asked to make a diagnosis again after presenting the OCTA images. The OCTA images include four images (superficial retina, deep retina, retinal pigment epithelium, and choriocapillary level; Figure 1). Sensitivity and specificity were calculated and compared between the 2-step results (before and after OCTA) and the gold standard of ICGA. Differences in diagnoses between the two graders were also compared to evaluate the diagnostic accuracy between the experienced and nonexperienced retinal doctors. Images with false-positive and false-negative results were evaluated and compared to identify the limitations of OCTA for the diagnosis of PCV.

Statistical analyses were performed with PASW Statistics for Windows, Version 18.0 (SPSS Inc., Chicago, Illinois, USA), using McNemar chi-square test. *P* < .05 was considered statistically significant.

## 3. Results

Among the 50 eyes, 31 were confirmed to have PCV and 19 to not have PCV by ICGA (11 with occult CNV, four with classic CNV, one with mixed type, two with a retinal angiomatous proliferation, and one with drusenoid PED). In comparison, an average of 32 eyes were diagnosed as PCV and 18 as non-PCV by the two graders. An average of 87.5% (28/32) were true positive, 12.5% (4/32) were false positive, 83.4% (15/18) were true negative, and 16.6% (3/18) were false negative.

The sensitivity increased from 69.5% (71% and 68%, resp.) to 90% for both graders from step 1 to step 2 after OCTA (Table 1). Figure 1 shows multimodal images of a true-positive PCV case for both graders. A mild decrease (from 84% to 74%) in specificity was noted for the senior retina specialist after OCTA due to an increased false-positive rate. However, for the retinal fellow, the specificity improved from 68% to 84%. Overall, the sensitivity significantly improved after OCTA (*p* = 0.046); however, the specificity did not (*p* = 0.856).

Six PCV cases (three each for the retina specialist and fellow) were misdiagnosed as being non-PCV (false negative) after providing OCTA images, all of which had unclear or insignificant BVNs in the OCTA images. Therefore, we further classified all of the PCV cases into two groups based on whether or not they had clear and obvious BVNs in the OCTA images. The results showed that 70.9% of the PCV cases (22/31) had clear or obvious BVNs and that this feature was the most sensitive to make an accurate diagnosis (sensitivity 97.5%). On the other hand, 29.1% of the PCV cases (9/31) had insignificant BVNs, all of which had a lower sensitivity of 72.5%.

Among the 11 cases of occult CNV, an average 27.2% (four for the retina specialist and two for the fellow) were overdiagnosed as PCV after OCTA. Other false-positive cases included one mixed-type CNV and one classic CNV. All false-positive cases had a BVN-like shape in OCTA.

## 4. Discussion

Our results showed that after providing color, FA, and cross-sectional SD-OCT images, OCTA increased the sensitivity of a diagnosis of PCV from 69.5% to 90% (*p* < 0.05). This increase in sensitivity was mainly due to the presence of BVNs in OCTA. Without manually adjusting for the segmentation line, BVNs were found in 70% and polyps in only 42% of our 31 cases with PCV. Unlike BVNs that resides within the Bruch's membrane, polyps are located on a more anterior and variable plane above BVNs and are identified in OCTA in less than half of cases [10]. In addition, the low flow of polyps and hence low signal in OCTA can decrease the detection of polyps [10]. With manual adjustments for the segmentation line, the polyp detection rate can be as high as 85%, whereas the detection rate of BVNs remains around 70% in OCTA compared to ICGA [11]. Therefore, BVNs are a more ready and critical feature than polyps for the diagnosis of PCV in OCTA.

**Figure 2:**
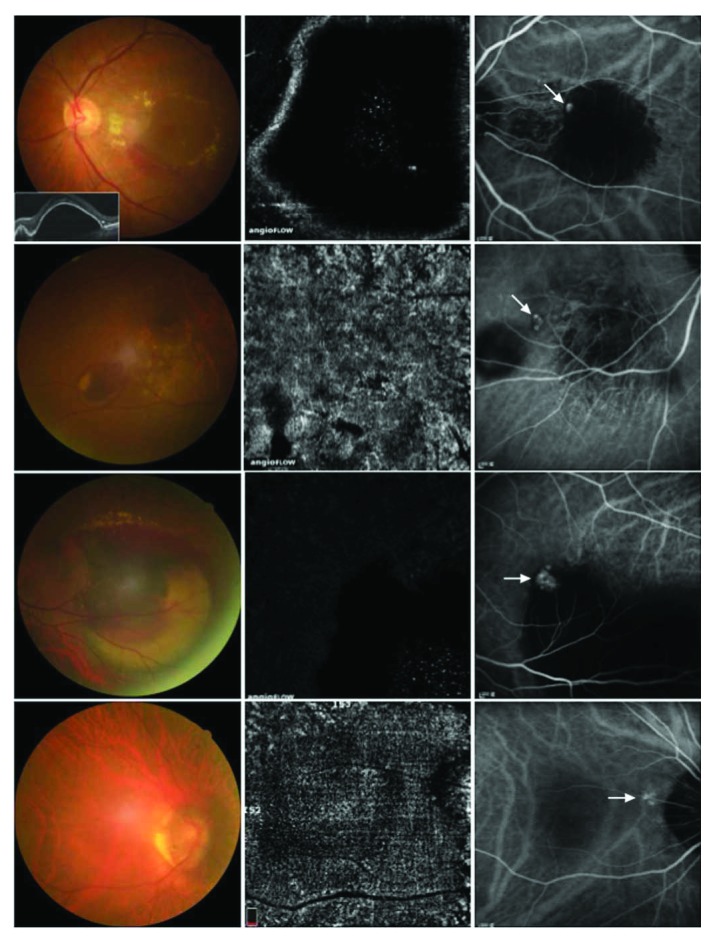
Four limitations of optical coherence tomography angiography (OCTA) for a diagnosis of polypoidal choroidal vasculopathy. Left: color fundus; middle: OCTA; right: indocyanine green angiography. The first row: large pigment epithelial detachments (PEDs). The second row: extramacular polyps with hemorrhage or exudation. The third row: massive organized blood and exudation. The fourth row: polyps without significant branching vascular nets.

**Figure 3:**
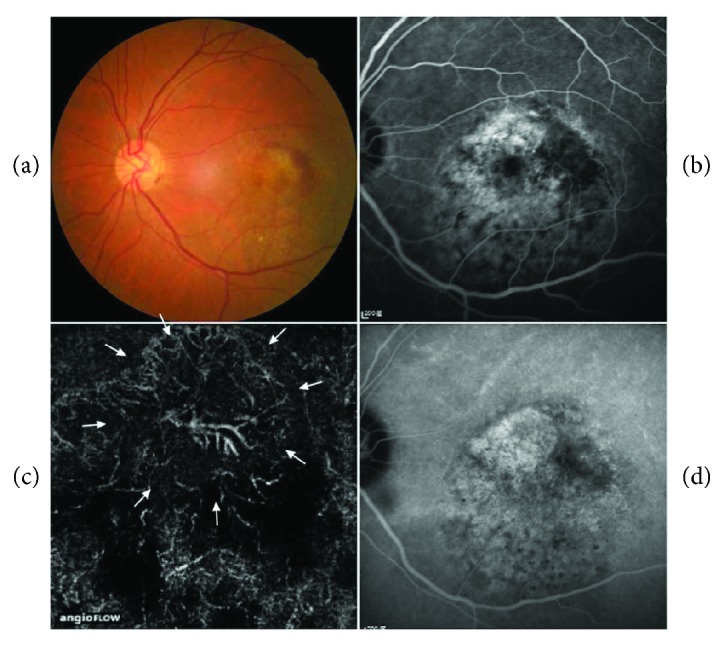
Occult choroidal neovascularization (occult CNV) could be misdiagnosed as idiopathic polypoidal choroidal vasculopathy by optical coherence tomography angiography (OCTA). Color fundus image showed a retinal hemorrhage (a), and fluorescein angiography showed an occult CNV leakage pattern (b). OCTA revealed a branching vascular net-like shape (arrows) (c). However, indocyanine green angiography did not detect any polyps (d).

In addition, if OCTA does not show BVNs, the diagnosis will be possibly undetermined, especially for cases with an organized hemorrhage, large PED, extramacular polyps, and polyps without BVNs as in our study (Figure 2). On the other hand, polyps can regress after anti-VEGF treatment, photodynamic therapy, or a combination of therapies [12]. In the false-positive cases with discernible vascular nets in OCTA but no polyps in ICGA, 75% (6/8) of the cases had previously received intravitreal injections of anti-VEGF, and two of six patients had a history of PCV in their fellow eyes.  Figure 3 shows an occult case of CNV misdiagnosed as PCV after OCTA because of a BVN-like structure at the choroidal level. Among these six cases, three had shallow irregular PEDs with a double-layer sign, two had a large PED, and one had hemorrhagic PEDs. Therefore, the possibility of regressed polyps in these patients cannot be ruled out due to previous treatments [13].

**Figure 4:**
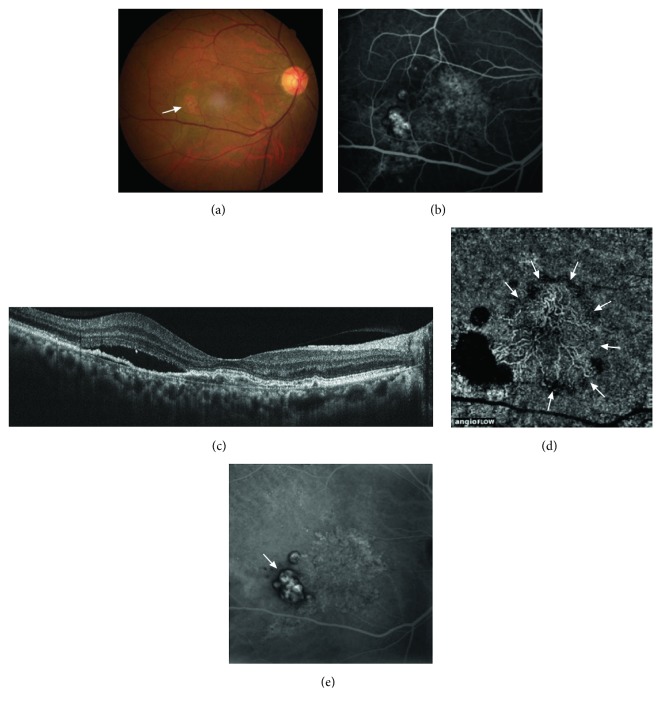
True-positive case of polypoidal choroidal vasculopathy (PCV). Subretinal orange nodules in a color image (arrow) (a), localized pigment epithelial detachments (PEDs) with delayed leakage of an occult choroidal neovascularization pattern in fluorescein angiography, (b) and subretinal fluid in spectral-domain optical coherence tomography (c) were noted. Optical coherence tomography angiography showed branching vascular nets (arrows) with surrounding PEDs at a choroidal level, but no significant polyps (d). Indocyanine green angiography confirmed polyps beneath the PEDs (arrow) and the diagnosis of PCV (e).

**Figure 5:**
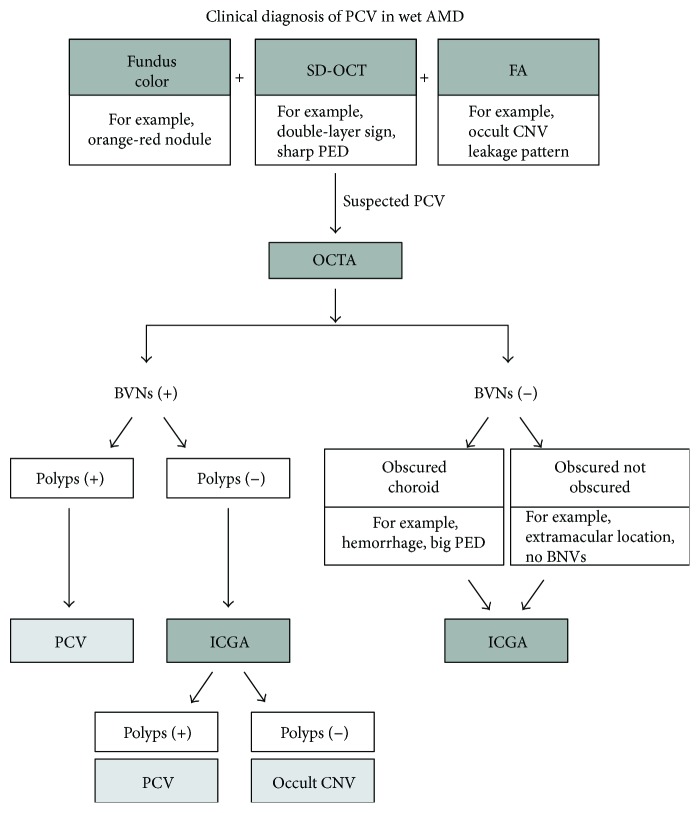
Clinical diagnosis of idiopathic polypoidal choroidal vasculopathy (PCV) in age-related macular degeneration (AMD) with optical coherence tomography angiography (OCTA). If the patient is suspected of having idiopathic polypoidal choroidal vasculopathy (PCV), OCTA is the next exam. With obvious branching vascular nets (BVNs) in OCTA, further polyp detection is the next sign for a diagnosis of PCV; if not, further indocyanine green angiography (ICGA) is required for cases without BVNs or further polyps in OCTA. FA: fluorescein angiography; PEDs: pigment epithelial detachments; SD-OCT: spectral-domain optical coherence tomography.

As shown in a typical case (Figure 4), several features help to diagnose PCV, including orange-red nodules in color photography, an occult CNV leakage pattern or serous PED in FA, and a double-layer sign with a sharp elevation in PED in cross-sectional SD-OCT. Salvo et al. used SD-OCT to diagnose PCV and reported sensitivity and specificity rates of higher than 90% [6]. However, they included only patients with PCV and occult CNV, while we included other subtypes of AMD and thus had lower sensitivity of 69.5% and specificity of 76%. Several features help to diagnose PCV as shown in the typical case in Figure 4. After OCTA, the sensitivity was increased to 90% based on BVNs. We therefore propose a diagnostic flow chart for PCV as shown in Figure 5. By fundus color, FA, SD-OCT, and additional OCTA, at least 42% (positive of both polyps and BVNs in OCTA in our series) of the suspected patients with PCV did not need to undergo ICGA.

Dansingani et al. also reported that in the eyes with pachychoroid features and shallow irregular PEDs, OCTA had a greater diagnostic value for type 1 neovascularization than FA and ICGA [14]. Furthermore, OCTA has also been reported to identify treatment-naive quiescent CNV to guide return visits and decisions regarding treatment [15]. Therefore, OCTA may be a useful follow-up tool for silent or active type 1 CNV.

There are several limitations to this study, including the small number of patients, autosegmentation of OCTA was not perfect without manual adjustments, and some (60%) cases had been treated before, which increased the difficulty of the diagnosis of PCV. However, we believed that the OCTA images were more representative of daily clinical practice and that postimage manual adjustments or processing was not available before the introduction of the automated retinal layer segmentation algorithm. [16]

In conclusion, by identifying BVNs in OCTA at a choroidal level, the sensitivity of diagnosing PCV with color, SD-OCT, and FA images increased by 20%. Future studies should investigate how well OCTA can identify polyps with more sophisticated analysis of imaging data in a cohort of patients with AMD and increase the diagnostic accuracy of PCV compared to ICGA.

## Figures and Tables

**Table 1:** Sensitivity and specificity before and after optical coherence tomography angiography (OCTA) in the diagnosis of polypoidal choroidal vasculopathy.

	Sensitivity			Specificity		
	Retina specialist	Fellow	Average	Retina specialist	Fellow	Average
Before OCTA	71%	68%	69.5%	84%	68%	76%
After OCTA	90%	90%	90%	74%	84%	79%
*p* value	^∗^0.046			0.856		

^∗^ *p* < 0.05.
